# Spatial Niche Segregation of Sympatric Stone Marten and Pine Marten – Avoidance of Competition or Selection of Optimal Habitat?

**DOI:** 10.1371/journal.pone.0139852

**Published:** 2015-10-07

**Authors:** Anna Wereszczuk, Andrzej Zalewski

**Affiliations:** Mammal Research Institute, Polish Academy of Sciences, Białowieża, Poland; University of Sydney, AUSTRALIA

## Abstract

Coexistence of ecologically similar species relies on differences in one or more dimensions of their ecological niches, such as space, time and resources in diel and/or seasonal scales. However, niche differentiation may result from other mechanisms such as avoidance of high predation pressure, different adaptations or requirements of ecologically similar species. Stone marten (*Martes foina*) and pine marten (*Martes martes*) occur sympatrically over a large area in Central Europe and utilize similar habitats and food, therefore it is expected that their coexistence requires differentiation in at least one of their niche dimensions or the mechanisms through which these dimensions are used. To test this hypothesis, we used differences in the species activity patterns and habitat selection, estimated with a resource selection function (RSF), to predict the relative probability of occurrence of the two species within a large forest complex in the northern geographic range of the stone marten. Stone martens were significantly heavier, have a longer body and a better body condition than pine martens. We found weak evidence for temporal niche segregation between the species. Stone and pine martens were both primarily nocturnal, but pine martens were active more frequently during the day and significantly reduced the duration of activity during autumn-winter. Stone and pine martens utilized different habitats and almost completely separated their habitat niches. Stone marten strongly preferred developed areas and avoided meadows and coniferous or deciduous forests. Pine marten preferred deciduous forest and small patches covered by trees, and avoided developed areas and meadows. We conclude that complete habitat segregation of the two marten species facilitates sympatric coexistence in this area. However, spatial niche segregation between these species was more likely due to differences in adaptation to cold climate, avoidance of high predator pressure and/or food preferences by both species than competitive interaction between them.

## Introduction

According to Gause’s principle [1], ecologically similar species sharing a niche cannot occur in the same area; one of the interacting species will be excluded [2], directly influencing community structure. Coexistence of two such species depends on sufficient niche differentiation which leads to inter-specific competition being weaker than intra-specific competition [3]. Coexisting species should differ in at least one or more dimensions of their ecological niches, such as resources, space or time, in diel and/or seasonal scales [4–7]. First, each species may specialize on distinct resources or respond in a different way to competitors [5, 8, 9]. Second, they may use resources or avoid enemies at different times [10–13], or thirdly, in different spaces [14, 15]. Such ecological niche differentiation is often assumed to be the result of past competitive interactions between species (the ghost of competition past) [16, 17]. Alternatively, a species’ niche differentiation may reflect differences in a multitude of requirements of the species which do not relate to past competition. In both cases, reducing current competitive interaction between species would enable them to coexist, however the reasons for and effects of niche separation are not the same. The first scenario assumes evolution through competition which enforces ecological differences between species to enable coexistence. In this case subordinate species, for example, often occupy less productive habitat at a low density [18, 19]. The second scenario assumes that closely related species have evolved separately (without competitive interaction) in different areas and may develop ecological differences as a result of adaptation to different biotic and abiotic conditions (e.g. climate conditions) [20, 21]. In such situations both species may occupy different but optimal habitats for each species and reach high densities in these habitats. Therefore, understanding why closely related species use different niches is a step toward elucidating the mechanisms behind coexistence.

Of the three types of niche differentiation, temporal niche partitioning between species is observed less often and usually occurs in situations where alternative mechanisms of coexistence cannot operate [22]. The presence of competitors can influence activity patterns, especially when similarity of other niche dimensions (like spatial niches and food resources) are high [5, 23]. Subordinate competitors may change their activity patterns thus allowing coexistence through a shift of their temporal niches [12, 24, 25]. In most cases, however, coexistence between closely-related species is possible by one species switching its habitat use towards areas where competitive interactions are reduced. This localized habitat partitioning could facilitate coexistence of the species in the same landscape; if one species predominantly uses space largely unoccupied by another, then the probability of occurrence of one competing species can be negatively predicted by the occurrence of the other [15].

Coexistence due to spatial niche partitioning can be facilitated in heterogenous landscapes with a mosaic of habitat patches of various specific biotic and abiotic conditions which provide appropriate resources for each species [3, 26, 27]. A heterogeneous landscape can also be created by human alteration of natural vegetation into open fields or agricultural and urban areas. Occupation of patches of such anthropogenic habitat by a species is often associated with disadvantages such as higher mortality risk from direct persecution, conflicts with domestic pets [28], and collisions with cars [29–33]. Despite these challenges, some species have adapted to anthropogenic habitats for various reasons, e.g., lower predation rates, reduced competition, or lower parasite abundance [34, 35]. Species also colonize urban and rural habitats because the availability of anthropogenic food is often higher and with less annual and seasonal variation than in natural habitats [36–39]. This stable year-round food supply may lead to better body conditions and shorter activity time periods in anthropogenic habitats which may consequently increase survival of the species. Therefore, in some conditions urban and agricultural landscapes may act as optimal habitat for species which can coexist with humans.

The stone marten (*Martes foina* Erxl. 1777) and pine marten (*Martes martes* L., 1758) are morphologically and ecologically similar species [28, 40, 41]. They are medium sized mustelids with a long, thin body shape. The body weight of both martens varies from 0.8–1.4 kg for females and 0.8–2.5 kg for males [42], however, this varies greatly with climate conditions and often contrary to Bergmann’s rule–martens are smaller in colder climates [43]. Where they co-occur the stone marten is usually slightly larger than the pine marten [44], which may position this species as dominant over the pine marten. Both species have a broad, opportunistic diet [45, 46]. The stone marten consumes mainly fruits, small mammals, birds and insects, whereas small mammals, birds, insects, plant material and carrion are the most important food categories in the diet of pine marten [47–51]. In areas of sympatry, the diets of both martens overlap extensively [52]. Despite their high similarity, these martens occur sympatrically over much of Europe. The pine marten occurs in North and Central Europe as well as in the Mediterranean region, but it is absent from large areas of Spain and Portugal [53]. The stone marten occupies areas from the Mediterranean region, through Portugal and Spain, to central and eastern Europe; it is absent from Scandinavia and northern European Russia [53]. There is little information about factors facilitating their coexistence; however, it is probably promoted in part by separation of their temporal and/or spatial niches which allows for utilization of different resources (food or resting sites). Data about temporal niche separation between both species is scarce in the literature; sources suggest that both species were active mostly from dusk to dawn, but activity patterns vary by season and sex [54, 55]. Small shifts in activity rhythms of stone and pine martens were observed in sympatry which may facilitate coexistence [56].

Both martens exhibit great adaptive plasticity and inhabit a wide range of habitats [46, 57], yet forests are crucial for both species [58–61]. In sympatric areas of Central Europe, the pine marten occurs in various types of forest, whereas the stone marten occurs in human-impacted habitats adjacent to forests: woodless areas, mosaic of forest and agricultural land and human settlements (cities and villages) [28, 60, 62–64]. In southern Europe, in areas where the two species are sympatric, stone martens use mosaics of forest and field patches [9, 65]; in areas where the pine marten is absent, the stone marten utilizes forests [66, 67]. These geographical differences in habitat use suggest that the stone marten may avoid forest in Central Europe due to competition with pine marten [68]. Both species use the same habitat in different ways. Under the same environmental conditions, pine marten make more intensive use of three-dimensional space in the forest than the stone marten (both during foraging and resting), but avoided all artificial elements in the forest (railway tracks, roads, buildings or rubbish dumps) [28]. Stone marten foraged more often in brushwood and piles of wood, visited logged areas and garbage dumps, and explored woodless areas and inhabited houses [28]. These studies were conducted in a mosaic of forest and agricultural areas with a relatively large number of villages, and in most cases top predators (e.g., lynx, *Lynx lynx*; wolf, *Canis lupus*) were absent or reduced. There are no data on habitat preferences of both species in large natural forests with few human settlements and a rich community of top predators [61].

The three main niche axes (space, time and resources) of the stone and pine martens seem to be similar and they occur in sympatry over a large area in Central Europe. Thus, in sympatry, we predict segregation of niches in space, manifested through differences in habitat preferences or in time, manifested via differences in diel activity patterns. The specific aims of this study were to 1) analyze differences in body size and condition of stone and pine martens; 2) analyze temporal and spatial niches of these species where they co-occur, and 3) compare seasonal variation of activity duration to analyze differences in adaptation to cold climate. To this end, we compare body size, activity and habitat selection of stone and pine martens in Białowieża Forest, eastern Poland, which comprises a large forest complex with patches of natural open habitats as well as patches of human-altered habitats (meadows or villages located inside of the forest), situated at the northern edge of the range of the stone marten.

## Materials and Methods

### Ethics Statement

All capture and handling procedures of the martens were approved by the Ministry of the Environment, Białowieża National Park and the Local Ethics Committee for Animal Experiments at the University of Białystok (no: Dlł-756/16/98; DL.gł-6713-21/35088/11/abr; DL.gł-6713-14/18806/11/abr; nr 2011/9). We acquired permission from private land owners to conduct study at their properties. The field studies did not involve endangered or threatened species.

### Study Area

This study was conducted in the Białowieża Forest of north-eastern Poland (52°43’N, 23°54’E). Białowieża Forest is a large primeval woodland covering over 1250 km^2^, in which old-growth forest areas are dominated by oak-lime-hornbeam stands comprised of hornbeam *Carpinus betulus*, oak *Quercus robur* and lime *Tilia cordata*, as well as scattered spruce *Picea abies*. Two other main forest types are mixed conifer (dominated by spruce and pine *Pinus silvestris*) and ash-alder (dominated by black alder *Alnus glutinosa* and ash *Fraxinus excelsior*). The forested portion of the study area (56.49 km^2^) includes the strictly protected Białowieża National Park (BNP) as well as the adjacent exploited forests. Old-growth forests of BNP are characterized by trees of various ages with a mean age of 130 years, the presence of snags and downed logs of large diameter, and small gaps in the canopy. The BNP has not been exploited for timber, and other human uses are restricted to the southwestern part. The local carnivore community includes the wolf, lynx, red fox *Vulpes vulpes*, badger *Meles meles* and the raccoon dog *Nyctereutes procyonoides*, an invasive species. The developed area (4.21 km^2^) is composed of four villages: Białowieża, Pogorzelce, Teremiski, and Budy. Białowieża village is the largest; it has 1803 inhabitants and is a compact settlement. Inside the villages are old orchards and scattered groups of old trees. Villages are surrounded by meadows and valleys of small rivers or forest. The meadows also contain scattered small patches (from 0.03 to 45.68 ha, mean 5.12 ha) of regenerating various trees species, mainly birch *Betula pendula*.

The climate in the study area is transitional between Atlantic and continental types, with continental features dominant, and distinct cold and warm seasons. The minimum day-time temperature in the study period was -33.5°C (in January or February), the maximum day-time temperature was 33.4°C (in July). Snow cover persists for an average of 85 days and snow depth ranged from 0–63 cm.

### Trapping, Marten Morphometry and Radio Tracking

From April 1991 to December 2012, 20 pine martens (10 females and 10 males) and 27 stone martens (12 females and 15 males) were captured in live traps baited with chicken, eggs and honey. Traps were spaced regularly across the entire study area, in all types of forests and inside villages. Captured martens were anesthetized using 15 mg/kg Ketamine and sexed, aged, weighed and measured to determine body length (without the tail). Martens were fitted with radio collars (AVM, Lotek or ATS) that weighed 12–25 g, and were less than 2% of the weight of the individual. The life span of the transmitter was 5–12 months. The marten’s age was determined according to tooth wear and two classes were distinguished: classified as ‘juvenile’ or ‘adult’ if less than or greater than 1 year of age, respectively. Martens were released after recovery at the site of capture. Five pine martens and 3 stone martens were tracked for only a short period or dispersed across the study area and were excluded from the analysis.

Body measurements were collected (after 1995) to assess differences in body size of both species and explore relations between body weight and body length (without the tail) as an index of body condition. To increase the morphological sample size, we included the measurements of additional individuals from road-killed martens (12 stone martens, 21 pine martens) collected in Białowieża Forest. To avoid bias of female body weight variation due to pregnancy, the females sampled in March and April were excluded from the analyses. A general linear model (GLM) implemented in R software (R 2.15.2) [69] was used to compare variation of body mass and length of stone and pine martens, using species and sex as variables, and including the interaction between the two. To analyze whether the relationship between body weight and body length varied significantly between species we used a linear model. Next stepwise simplification of the model was performed (we removed species from the model) and goodness of fit of these two models with and without species was assessed by comparing their residual sums of squares (RSS) with an ANOVA in both models. Body condition was analyzed by calculating body mass index (BMI = body weight (kg)/ body length (m^3^)) [70].

To measure activity rhythms, duration of activity and habitat selection we tracked collared martens using a receiver (Telonics or Yaesu, FT 817 ND) and H-shaped or 3-element Yagi antenna. The radio-collared martens were located by an observer on foot one or two times per day (once in daylight and/or night) at least four times per week. Bearings were obtained from a distance < 500 m, usually about 300 m in the forest and 200 m in the village. We plotted locations on 1:10,000 maps, then transferred the coordinates to ArcGIS 9.3 (Environmental Systems Research Institute, Redlands, California). In most cases the hand plotting on the map was more precise, instead of triangulation, especially in the villages as there are many easily distinguishable elements (e.g. houses, barns, roads). The mean location error in the forest was below 65 m (average 34 m) estimated based on 40 bearings obtained in a blind test. Besides obtaining one location per day, some individuals were monitored continuously for 4–24 h sessions and locations were taken at 15-min intervals to assess marten activity, from one to twenty sessions per individual. Martens were characterized as active when the signal frequently switched pulse amplitude, or inactive when signal pulse amplitude remained constant. We used all locations to analyze the activity patterns, but we only used one location with a minimum 2-hour interval in our analyses of habitat selection to reduce temporal autocorrelation among locations. We selected 2-hour intervals as martens may move from one location to another location within their home range during that time period, therefore we assumed that these locations were independent.

To estimate seasonal rhythms and daily duration of marten activity, all locations were pooled for 2-h periods in each season. For each 2-h period, fixes when martens were active were calculated as a percentage of all fixes for each individual. Weighted averages of marten activity were counted for every 2-h period, with number of locations for each individual in each period as a weight. The duration of activity in each 2-h period was estimated and summed for the day. The daily duration estimated from summed 2-h period activity correlated highly with estimates from 24-h tracking sessions [71]. The data were analyzed for 3 seasons: spring (16 March–15 June), summer (16 June–15 October) and autumn-winter (16 October–15 March). We used Generalized Additive Models (GAM) to determine the effect of hour, species and sex on activity rhythms of both marten species. A cyclic cubic spline smoother was used in nonlinear model to constrain the point at the end of the day is the same as that at the beginning (package ‘mgcv’ [72]). To compare duration of martens activity, linear mixed-effects model were used, with individuals as a random factor (package ‘nlme’; [73]). The best models were selected using AIC value and model averaging was performed with the dredge function with the ‘MuMIn’ package for R [74].

### Habitat Selection Analysis

Previous studies of stone and pine martens in Europe have highlighted the importance of vegetation type, openness and human-inhabited areas in habitat selection by these species [60, 62, 66, 68, 75, 76]. We selected a set of eight variables that were germane to our study as we hypothesized *a priori* that they could influence habitat selection of one or both of the marten species. We obtained vector data of forest types from The State Forests National Forest Holding (Information System of the State Forests SILP; [77]) at a 1:10000 scale. Based on this map we reclassified 15 forest types to 3 main types: deciduous forests (DCI), coniferous forests (CNI), and bog forests (BOG) (Table 1). The distribution of another five land cover types (meadows (MEA), rivers (RIV), open wetlands (WET), patches with tree regeneration (TRE) and developed areas (DEV)) were obtained from the Database of Topographic Object map (http://geoportal.gov.pl). We believe that a combination of these land cover variables will contribute to understanding habitat use and selection by both martens without topographic variables as the study area is mostly flat (134–186 m a.s.l.). The digital resource maps were obtained by merging habitat classes from the original database maps and revised by ground-truthing. All maps were prepared as a raster with 10 m resolution, and spatial analyses were carried out in ArcGIS 9.3 software, with Spatial Analysis and Hawth’s Analysis Tools extensions. Study area was determined with a 99% fixed-kernel home range, with a smoothing parameter h = 1200, calculated using all independent locations of all animals of both species [78,79]. To show habitat use by martens, we calculated the proportion of locations in each of the eight land cover types. Not all habitat types in the study area were used by either marten species. Therefore we selected a distance-based approach to estimate habitat selection and for each location we calculated the nearest distances to each habitat type and used in further analysis. We also selected a distance-based method over a composition-based method because this approach is less sensitive to spatial telemetry error and habitat misclassification [80]. Furthermore, linear habitat features (especially rivers) would not be adequately represented in compositional analysis. To represent availability of habitats, a total of 2000 random points were generated and for each random point the proportion of each location in each eight types of habitat and the nearest distances to each habitat type were also estimated. We calculated the distances from marten locations and random points to each habitat using Point Distance script from ET GeoWizards 9.9 implemented into ArcGIS 9.3.1 Model Builder. The distances of marten locations and random points to each habitat type were transformed using function ‘scale’ (scaling and centering of matrix-like objects; package ‘base’) [81, 82].

**Table 1:** Description of habitat variables used to estimate resource selection functions for stone and pine martens radio-tracked in Białowieża Forest, north-eastern Poland.

Variables	Description
DEV	Developed areas with compact settlements such as villages and towns
MEA	Open areas, mainly meadows, as well as arable lands, wastelands
WET	Open wetlands and river valleys
RIV	Medium sized rivers and small streams
BOG	Bog forests dominated by black alder *Alnus glutinosa*
TRE	Small patches of trees surrounded by open areas, scattered trees; average age of 30–40 years
CNI	Coniferous forest dominated by spruce *Picea abies* and/or pine *Pinus silvestris*
DCI	Deciduous forest dominated by oak-lime-hornbeam stands

Habitat selection was analyzed using resource selection function (RSF) for use-availability data for each species, and for each sex of both species separately. RSF is defined as any function that is proportional to the probability of selection of a resource unit, where habitat type is an attribute of a resource unit [82–84]. We estimated RSFs assuming the selection function took the exponential form [85, 86] and estimated model coefficients using logistic regression in a use-available design and nonparametric bootstrap standard error (B = 99). The RSF was estimated using equation:

$$\text{w}\left(x\right)\;=\;\text{exp}\left(\beta_{1}{\text{x}}_{1}+\;\beta_{2}{\text{x}}_{2}+\;\ldots \;+\;\beta_{\text{k}}{\text{x}}_{\text{k}}\right)$$ (1)

where β_k_ represented the selection coefficient for variables x_k_ in vector, **x**, of k covariates, and w(**x**) is the RSF. We based the RSF on the comparison of distances between telemetry locations of martens to every habitat type (used units, coded 1) and distances from random points to every habitat type (available units, coded 0). We conducted analyses in R 2.15.2, in package “ResourceSelection” [87]. We used Akaike’s Information Criterion corrected for small sample size (AIC_c_) and Akaike’s weight (wi) to select the most parsimonious model of marten habitat selection [88] using package “MuMIn” [74]. The minus sign of β coefficients means preference to habitat type, because it represents a shorter distance from marten locations to that habitat type when compared to the distance from random points (if location was situated in a given type of habitat, distance from this location to that habitat is 0). Positive values of coefficients means avoidance to given habitat type.

To generate maps representing the relative probability of occurrence for both martens, RSF predicted values were estimated from Eq 1 and linear stretch used to scale predicted values of the RSF between 0 and 1 (Eq 2). The linear stretch is a common transformation for image enhancement and interpretation [89, 90] and takes the form:

$$\hat{\text{w}}\;=\;\left(\text{w}\left(\text{x}\right)-{\text{w}}_{\text{min}\_}/\;{\text{w}}_{\text{max}}-{\text{w}}_{\text{min}}\right)$$ (2)

where w(x) is the product of Eq 1 and w_min_ and w_max_ represent the smallest and largest RSF values.

## Results

### Body Size

**Fig 1:**
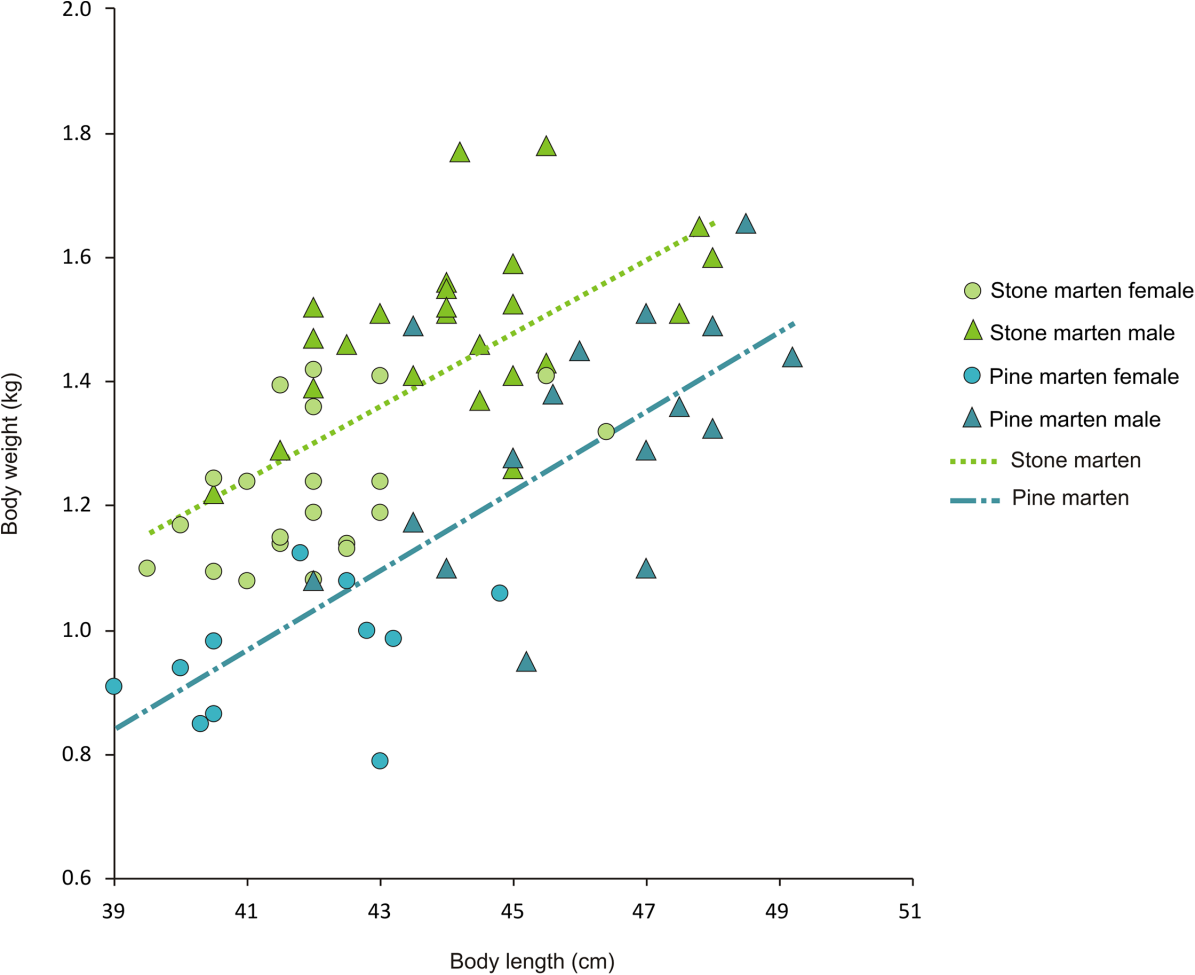
Relationships between body weight (BW) and length (BL) of stone and pine martens in north-eastern Poland. The regression equation for stone marten is BW = -1.0834 + 0.056*BL and for pine marten is BW = -1.656 + 0.063*BL.

We collected measurements and body weight of 46 individuals of stone martens (21 females and 25 males) and 27 individuals of pine martens (11 females and 16 males). The best models suggested that both sex and species were influential factors in the best model for body mass (S1 Table); males were heavier than females of both species (t = 6.43, P<0.001; Table 2) and pine martens were about 0.2 kg lighter than stone martens (t = -4.91, P<0.001; Table 2). Marten body mass was positively correlated with body length (t = 4.85, P<0.001; Fig 1). However, stone martens were significantly heavier than pine martens for the same body length. The comparison of models with and without nested species were significant (ANOVA, P<0.001); the model with nested species having a significant lower Residual Sum of Squares (RSS = 1.04) compared to the model without (RSS = 2.05). Stone martens weighed on average 256 g more than pine martens with the same body length. The mean body mass index (BMI) of stone marten was 16.94 (16.55 for females and 17.14 for males) and pine marten was 13.58 (13.28 for females and 13.44 for males).

**Table 2:** Variation of stone and pine martens body weight (kg) and length (cm).

Species	Sex	Body weight			Body length		
		Mean	SE	Range	Mean	SE	Range
Stone marten	Female	1.23	0.025	1.08–1.42	42.04	0.354	39.5–46.4
	Male	1.50	0.029	1.22–1.78	44.45	0.460	40.5–50.8
Pine marten	Female	0.96	0.030	0.79–1.12	41.70	0.535	39.0–44.8
	Male	1.32	0.057	0.95–1.65	46.10	0.575	42.0–49.2

### Temporal Niche Segregation

**Fig 2:**
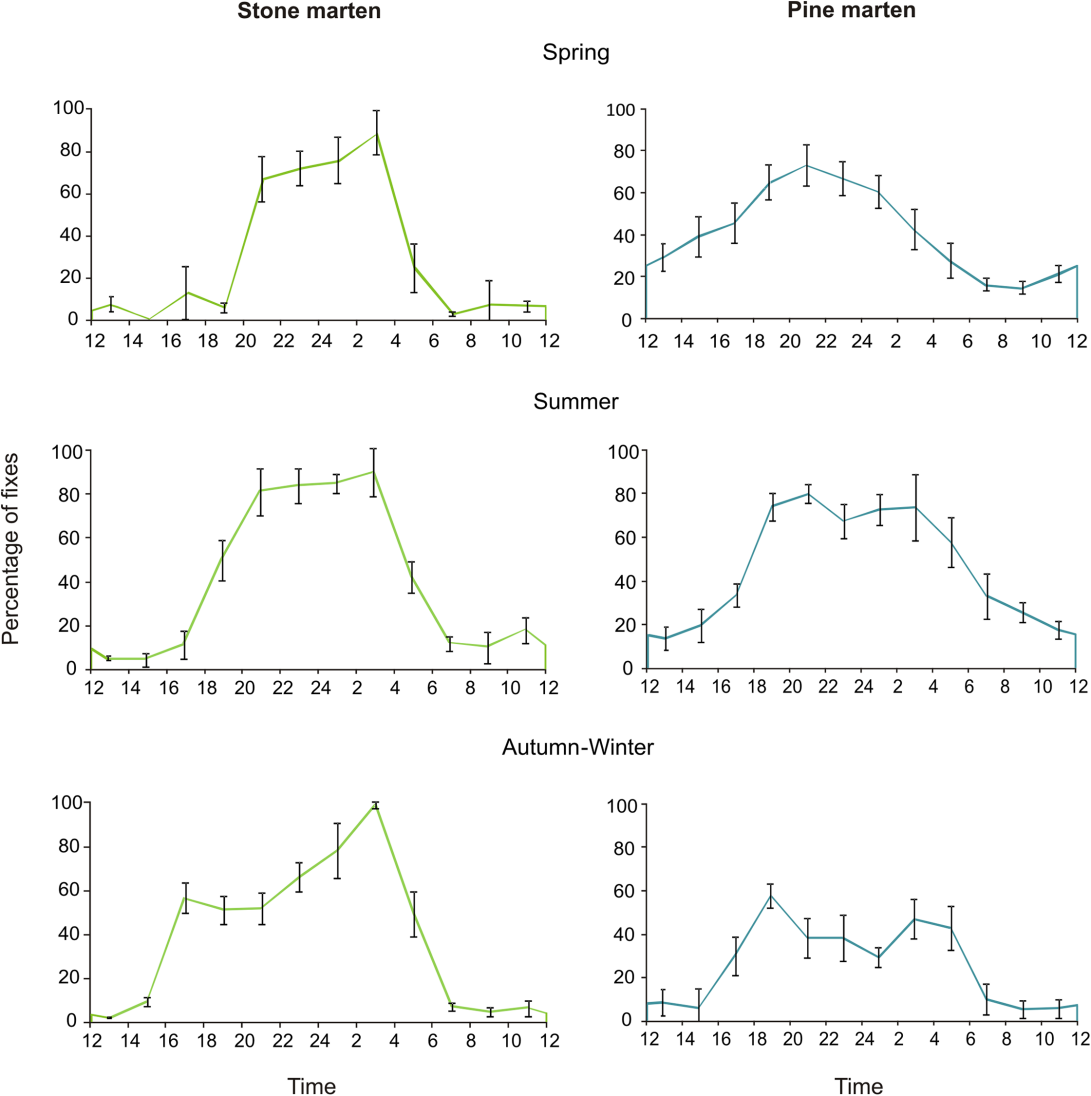
Seasonal variation of activity rhythms of stone and pine martens in north-eastern Poland.

We obtained 6 131 locations of 24 collared stone martens and 23 643 locations of 15 collared pine martens. Both marten species were mainly nocturnal; stone martens started their activity generally at 6 p.m. and finished it about 6:00 a.m. Pine martens became active earlier and were more frequently active during the day (Fig 2). The top model explaining variation in diel activity rhythms indicates importance of hour, season, sex and species (AIC_c_ = 29866.6; S2 Table). Differences between seasons were significant between all pairs: spring and summer (GAM; t = 6.89 P<0.001) and summer and autumn-winter (t = 26.60, P<0.001) and autumn-winter and spring (t = 17.43, P<0.001). Both sexes showed differences in activity rhythms (t = 5.05, P<0.001). There were also differences in pattern of diel activity rhythms between species (t = -2.24, P = 0.025). Pine martens were active more often in daylight hours than stone marten, especially in spring and summer. Furthermore, the peaks of activity for both species occurred in different periods of the night; stone marten’s activity was greatest at 4 a.m., in contrast to pine marten which activity peaked at 8 p.m. The activity of stone and pine martens differed between hours of the day (GAM; edf = 8.86, F = 784.8, P<0.001).

Throughout the year, stone marten females were active on average 8.16 h and males 9.25 h per day, whereas pine marten females were active on average 8.83 h and males 9.73 h per day (Table 3). The best models explaining duration of marten’s activity (AIC_c_ = 263.1; S3 Table) show the importance of species, season and the interaction of species and season. The duration of marten activity differed between spring and summer (t = 3.94, P<0.001) and between autumn-winter and spring (t = -2.68, P = 0.013), but there were no differences between autumn-winter and summer (t = 1.20, P = 0.24). Interaction between season and species were significant for all seasons. Both species were most active during summer (stone marten on average 8.73 h and pine marten 12.16 h), but pine martens decreased their activity in autumn-winter, where stone martens decreased activity in spring (Table 3). The top models predicting duration of martens activity with ΔAIC_c_ < 4 included additional variables–sex and interaction of sex and season (S3 Table). Male of stone martens were active longer in all seasons than females, also male pine martens were active longer than females, except in the summer, when females were active for 0.57 h longer than males (Table 3).

**Table 3:** Seasonal variation in duration of diel activity (in hours) of stone and pine martens calculated from radio-tracking data in north-eastern Poland.

Season	Stone marten				Pine marten			
	Female	SE	Male	SE	Female	SE	Male	SE
Spring	6.65	0.81	8.20	1.34	8.99	0.84	10.15	0.45
Summer	8.73	0.65	10.16	0.88	12.16	0.76	11.59	0.53
Autumn-Winter	9.11	0.62	9.40	0.62	5.33	0.83	7.46	0.79
**Average**	**8.20**	0.48	**9.57**	0.50	**8.79**	0.79	**9.37**	0.56

### Spatial Niche Segregation

**Fig 3:**
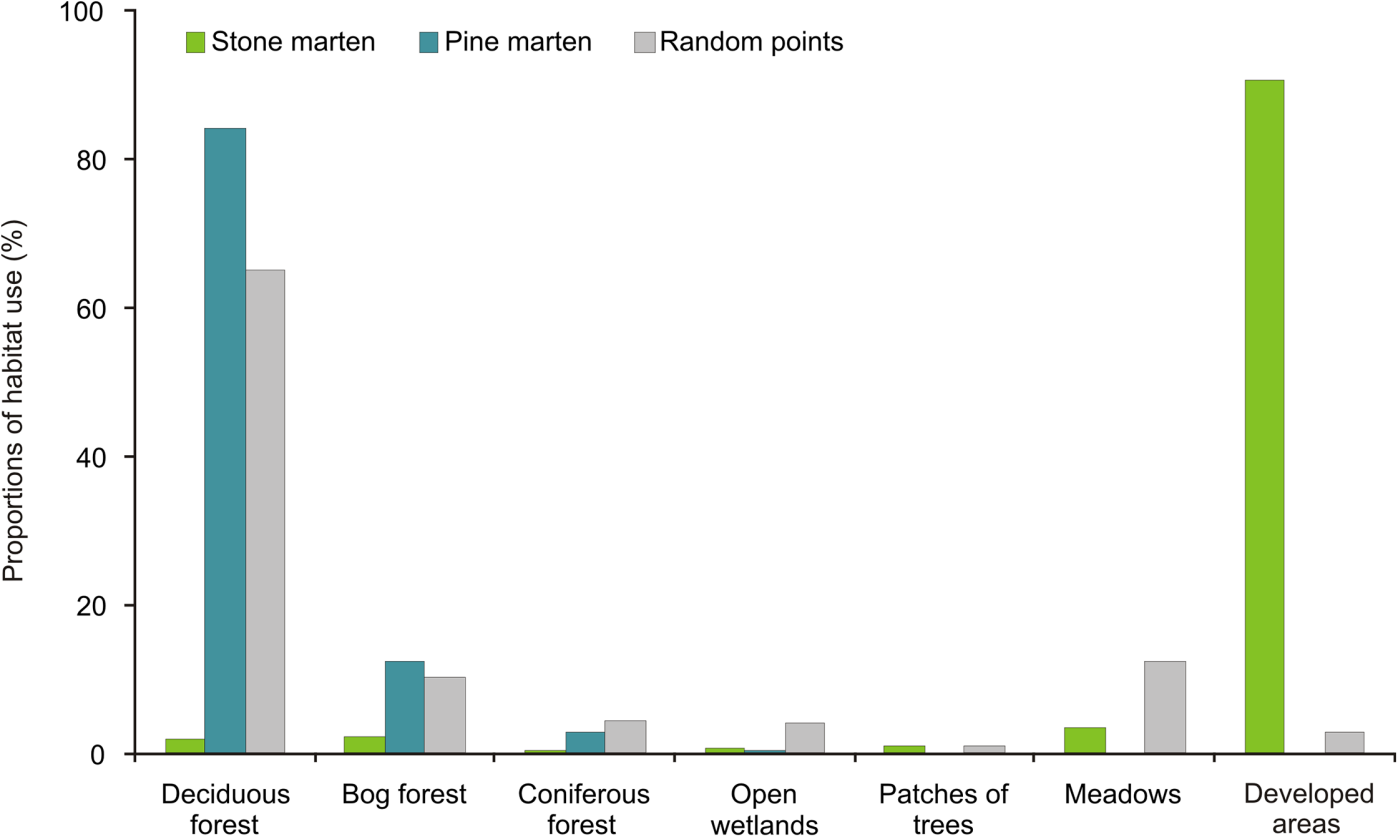
Frequency of habitat types used by stone and pine martens compared to the frequency of available habitat types calculated based on random points in the study area of Białowieża Forest.

In total, we obtained 2 556 independent locations for stone martens (10 females and 11 males) and 3 624 independent locations for pine martens (7 females and 6 males). Stone martens were located mainly in villages and occasionally in meadows and wetlands surrounding villages. Pine martens were found only in forests (deciduous, bog and coniferous forests) and never in other habitat types (Fig 3). Season did not influence habitat use in either species (GLM, t = 0.42, P = 0.68), thus further analysis was performed for data pooled for all seasons.

We observed differences in habitat selection between stone and pine martens. The top ranking model that best described resource selection by both marten species included all 8 types of habitat (Table 4). Stone marten strongly preferred developed areas (β = -56.69), and avoided meadows (β = 6.16; Table 5) and coniferous or deciduous forests. This species also preferred habitats near rivers or near open wetlands and small patches with tree regeneration. Pine martens preferred deciduous forest and also small patches covered by trees. Simultaneously, they avoided developed areas (β = 0.74) and meadows (β = 0.88; Table 5). Both sexes of stone marten demonstrated a strong avoidance of meadows, but males avoided them more than females (Table 5). Slight differences between sexes were observed in preferences of open wetlands and bog forests–females prefer them while males avoid these types of habitat. Males preferred villages slightly less than females, but they more often were found near rivers and small patches with tree regeneration. Both sexes preferred habitat close to rivers, but stone marten males were located closer to rivers. Female pine martens preferred deciduous forest and bog forest more than males, while males preferred coniferous forests and small patches covered by trees more than females. Female pine martens utilized habitats closer to the rivers than males. Male pine martens preferred open wetland whereas females were indifferent to this type of habitat (Table 5).

**Table 4:** Two sets of resource selection function (RSF) models for stone and pine martens ranked by Akaike’s information criterion scores (ΔAIC_c_) and AIC_c_ weights for candidate RSF models developed for stone and pine marten populations of north-eastern Poland.

No	Covariates	AICc	ΔAIC_c_	Weight
**Stone marten**				
1	DEV, RIV, TRE, WET, BOG, DCI, CNI, MEA	21915.9	0.00	0.775
2	DEV, RIV, TRE, BOG, DCI, CNI, MEA	21918.4	2.48	0.224
3	DEV, RIV, TRE, WET, DCI, CNI, MEA	21932.3	16.37	0.00
**Pine marten**				
1	DCI, TRE, RIV, WET, CNI, BOG, DEV, MEA	48724.8	0.00	1.00
2	DCI, TRE, RIV, WET, CNI, DEV, MEA	48815.4	90.67	0.00
3	DCI, TRE, RIV, WET, BOG, DEV, MEA	48964.9	240.12	0.00

**Table 5:** Coefficients (*β*) and standard errors (SE) of the best models for sets of habitat covariates in resource selection function (RSF) models for habitat selection of stone and pine martens in north-eastern Poland.

Covariates	Stone marten						Pine marten					
	Both sexes		Female		Male		Both sexes		Female		Male	
	*β*	SE	*β*	SE	*β*	SE	*β*	SE	*β*	SE	*β*	SE
DEV	-56.69	3.20	-74.93	5.48	-55.97	5.85	0.74	0.03	1.02	0.07	0.75	0.04
MEA	6.16	0.36	6.38	0.56	9.07	0.46	0.88	0.03	0.30	0.06	1.24	0.04
WET	-0.37	0.15	-3.56	0.28	2.92	0.29	-0.49	0.02	0.14	0.04	- 0.82	0.02
RIV	-0.73	0.11	-0.44	0.15	-1.91	0.22	-0.68	0.02	- 1.07	0.04	- 0.51	0.03
BOG	-0.11	0.03	-0.58	0.04	0.44	0.04	-0.23	0.02	- 0.48	0.05	- 0.10	0.03
TRE	-0.48	0.03	-0.16	0.05	-0.76	0.05	-1.45	0.03	- 1.46	0.06	- 1.66	0.05
CNI	0.61	0.04	0.26	0.05	1.29	0.07	-0.34	0.02	- 0.08	0.03	- 0.52	0.02
DCI	0.49	0.02	0.93	0.04	0.24	0.02	-2.15	0.11	- 3.51	0.40	- 2.24	0.11

**Fig 4:**
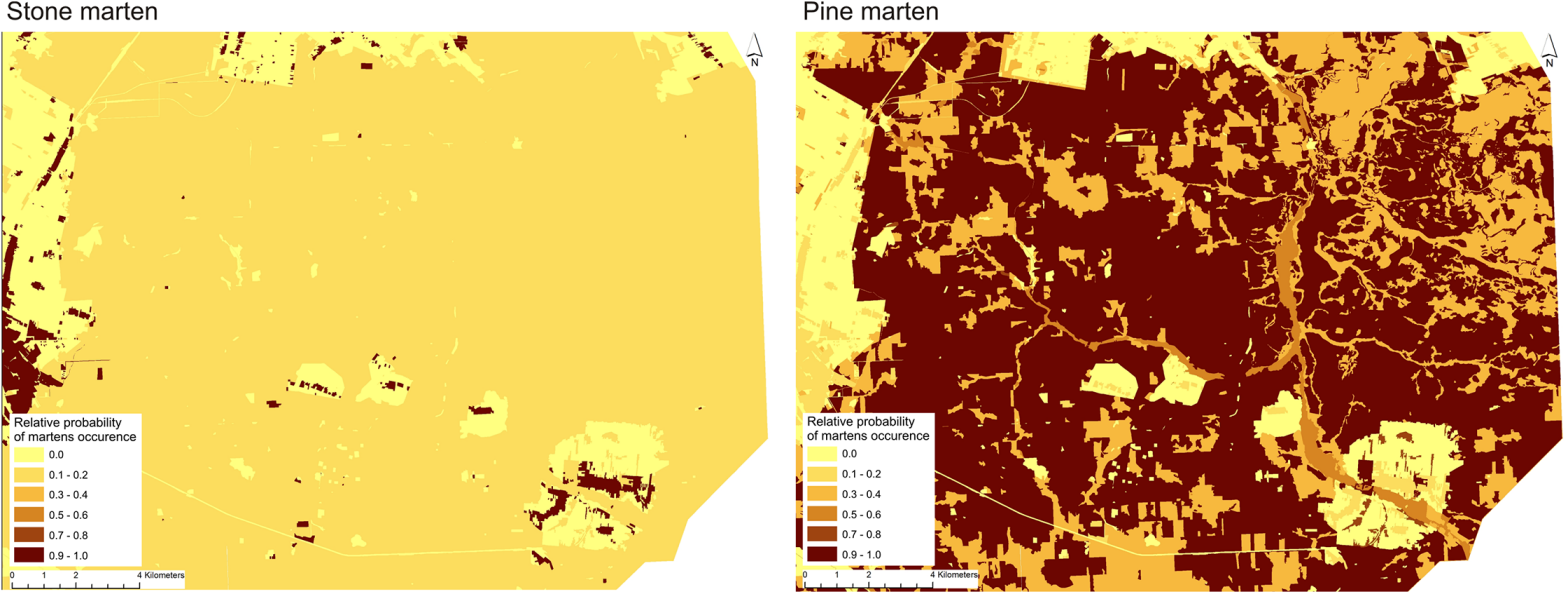
Stone and pine martens distribution in various habitat type in the area of Białowieża Forest predicted using resource selection functions (RSFs).

Habitats preferred by stone marten were avoided by pine marten and *vice versa*, and this was reflected on maps representing relative probability of occurrence of both marten species (Fig 4). Preferred stone marten habitat (developed areas) were very fragmented and surrounded by meadows and forests, which were strongly avoided. Availability of preferred pine marten habitat (various forest types) was high, including a broad homogenous area. Thus, Białowieża Forest constituted a large area with available habitats for pine marten and a few isolated “islands” (villages) utilized by stone martens surrounded by forest. In contrast, areas outside of the Białowieża Forest contained more habitats that were suitable for stone martens and adverse for pine marten.

## Discussion

Our results suggest a strong ecological niche segregation between the two marten species that facilitates their coexistence. Both species demonstrated almost complete separation of their spatial niches in all seasons, where the larger and potentially dominant species—stone marten—preferred human-settlements whereas the smaller pine marten preferred forests (especially deciduous forest). In addition, we found weak evidence of temporal niche segregation between the species. In all seasons, stone and pine martens were active mainly during the night, similar to other regions in Europe; however, stone martens were almost strictly nocturnal, while pine martens were facultatively nocturnal [91, 92]. The strictly nocturnal activity of stone martens might be related to human activity in villages and this species was more active at times of the lowest human activity (the peak of its activity was about 4 a.m.). Similarly, urban stone martens were completely nocturnal, presumably due to adaptations in their activity rhythms to avoid human encounter and reduce risk with car collisions [93]. Temporal niche segregation did not appear to play an important role in reducing interspecific competition between stone and pine martens.

We hypothesize that habitat segregation facilitates coexistence of stone and pine martens in Białowieża Forest. Stone martens inhabited mostly villages and avoided various types of forests and open habitats (e.g. meadows). Our findings about stone marten habitat use are corroborated with previous findings from Central Europe, where this species also occupied urban and rural areas [28, 93–95]. However, in southern Europe stone marten occur in a much greater variety of habitat types, including cork oak woodlands, rocky areas, fields, pastures and wooded farmlands [61]. In the Białowieża Forest pine martens, selected different types of forest, mainly deciduous forests, as well as scattered tree patches and wetlands, but avoided meadows and villages. This is similar to other sites in northern and central Europe where pine martens occur in old-growth forests or mature coniferous plantation [76, 96–98]. In southern and western Europe, pine martens also inhabited a mosaic of woodlands, cultivated fields, grasslands and orchards [28, 57, 62, 64, 99]; in these studies the densities of this forest-dwelling species were lower and home ranges were larger in fragmented forest habitats [59]. In our study area, the density of both marten species in preferred habitats was very high ([59], Wereszczuk unpublished data). Our data for the first time show nearly complete spatial niche segregation between these martens, in contradiction to other studies where both species occupied similar habitats [28, 56, 100]. Despite the fact that stone and pine martens are capable of inhabiting the same habitat types in sympatric areas [61], in north-eastern Poland within a large forest complex they avoided habitats occupied by the other marten species.

One possible and obvious explanation of the near complete habitat niche segregation between these species is competitive interactions between stone and pine martens. In competitive interactions, larger species are typically dominant over smaller ones, excluding the smaller species from the landscape or at least reducing their density [101]. In our study area, the stone martens were significantly bigger than pine martens. Therefore, if we assume that interspecific competition affected almost complete separation of the spatial niches of both martens, we should expect that stone marten could potentially dominate over pine marten and choose the most optimal habitat. Indeed, some studies have suggested that stone martens can be a dominant competitor over pine martens in mountainous agricultural-forest mosaic on Iberian Peninsula [56]. Other studies, however, suggest that pine martens have excluded stone martens from natural habitats and only in areas where pine martens are absent will stone martens inhabit more natural habitats such as mature oak woodlands [63, 99]. Stone and pine martens can also coexist without spatial segregation with slight differences in diet and activity rhythms [56]. These types of coexistence interactions of the two species in one area may relate to differences in habitat structure and/or climatic conditions in various sites over the sympatric range of martens. The question therefore is why the larger and potentially dominant stone marten did not occur in natural forest habitats?

Beside the competitive interaction, an alternative explanation of the habitat preferences and segregation by both martens may be their different morphological and behavioral adaptation to cold climate. Generally mustelids, including both marten species, exhibit poor physiological adaptations to cold climates. The metabolic costs of mustelids are high, with their long and thin body shape, and thermoregulation is difficult to maintain without the ability to store body fat. Martens and other small mustelids require behavioral adaptations to help balance their metabolic costs, for example behavioral thermoregulation. Pine martens reduce activity during autumn-winter to reduce exposure to low temperatures and energy loss [55, 71]; this species was active 13 h per day when ambient temperatures were 25°C and curtailed activity to 2 h per day in temperatures of -20°C [71]. By limiting activity, pine martens reduced distances of daily movements for foraging [49]. During these shortened periods of activity, pine martens increased their foraging efficiency by hunting bigger prey or scavenging on ungulate carcasses [71]. The reduced time of exposure to low temperatures was possible only when animals could retreat to well-insulated resting sites. In temperate deciduous forests, most resting sites used by pine martens were located up in trees (93% of resting sites) in cavities or nests, mainly squirrel nests [102]. These sites are well insulated, and also protected martens from predation.

In contrast, the stone marten used different behaviors to reduce the cost of thermoregulation in winter. In our study, the average autumn-winter duration of activity was similar to that in summer and longer than activity duration of pine marten in autumn-winter. Similarly, in other studies there was also no evidence of decreased activity duration in winter by stone martens [103]. Furthermore, in farmlands with a mosaic of forest and fields or urban habitats, stone martens used arboreal resting sites relatively infrequently (>3%) compared to the pine marten [91, 103–105]. In Portugal, only 35% of forest-dwelling stone marten rest sites were arboreal [53]. The stone marten is less arboreal as a species than the pine marten [28], which reduces the prospects of stone martens selecting better insulated and safer resting sites up in the trees in winter. Instead of using cavities or squirrel nests during cold days, stone martens frequently used resting sites in buildings—houses, barns, stables ([91, 104, 105], A. Wereszczuk unpublished data). Stone martens also shift from using uninhabited buildings during the warm season to attics of inhabited buildings in the cold season [105]. In our study, stone martens also stayed inside barns for few days where they can prey on rodents (Wereszczuk pers. obs.). Inside the buildings the temperature is higher than ambient temperature [106] and stone martens are protected from heat loss, thus can be active for a longer time to find sufficient food. These differences in behavior of both martens may relate to some morphological differences between them. For example, stone marten fur is less dense than that of pine marten and stone marten feet are hairless. Both of these morphological differences increase heat loss of stone marten.

The differences in behavioral thermoregulation between stone and pine martens may be a consequence of different places where these species evolved and differences in the history of the colonization of Europe by both species. Pine martens arrived in Central Europe during the Allerød, coinciding with the recolonization by birch and pine woods [107]. It is known from late Pleistocene from central and western Europe and by postglacial times it had spread to Scandinavia. The presence of pine martens has been used to indicate coniferous forests and cold climatic conditions [41]. Stone martens evolved in subtropical forest of Middle Asia and started colonizing Europe from the southeast [107, 108]. The colonization of Europe by stone martens coincided with the colonization of the area by humans [41, 107, 109]. Therefore, it may have better morphological or behavioral adaptations to warm climates. The current geographic ranges support differences in adaptations of both species to severe winters. The northern European limit of the range of stone marten is Denmark, Latvia and Estonia, and the eastern limit is the Ukraine, Belarus and western Russia [53]. The pine marten range extends farther north and it occurs throughout Scandinavia and also a large area of Russia to the Ural [53].

If low temperatures are the only explanation of spatial niche segregation of both martens, we would expect stone martens to utilize various forest types from spring to autumn, as the ambient temperature is much higher in these seasons. Seasonal variation in habitat use in a variety of species has been observed, generally with a wider range of habitat types used in summer than in winter (e.g. [110]). However, the most distinct habitat segregation between competitive species should be observed during the reproductive period when larger species use habitats with higher food abundance (e.g. [111]). We did not observe any seasonal variation in habitat use by either marten species. An alternative, but not mutually exclusive, explanation of strong preference for villages by stone marten could be either high food abundance or lower predation pressure in human-associated habitats. In villages, fruits constituted the most important food type for stone marten (M. Czernik, unpublished data) and the abundance of fruits in villages is very high from the end of spring to the end of autumn, compared to the abundance of fruits in the forest. Furthermore, living in the vicinity of humans also allowed the stone marten to access other food types, such as human food waste deposited in compost sites or bins, poultry and eggs, and pet food. These food types are stable and available throughout the year. High food abundance affected, for example, marten activity and body condition [49]. Indeed, in spring and summer stone martens were active for shorter periods than pine martens which suggest higher food abundance in villages. We also found that stone martens with similar body lengths were heavier than pine martens; this may relate to two factors. First, body proportions in both marten species could be different, which affects relationship between body weight and length. However, comparisons of the relationship between the body weight and length from other study sites did not show any differences between pine and stone martens (A. Wereszczuk in prep.). For example in sites where both species used the same habitats, the weights of individuals with same body length and BMI were similar in both species (15.42 for stone marten and 15.35 for pine marten in Switzerland and 13.07 for stone marten and 12.50 for pine marten in Luxemburg; [44, 93]). The second explanation is that the stone martens inhabiting villages accumulate more fat reserves than pine martens from the forest. The better body condition of stone marten suggests that the food abundance in the villages was higher than in the forest. The higher food availability is the most likely explanation for preference of villages by stone martens throughout the year. The higher body weight due to fat reserve carries the risk of impaired access to resting sites, especially arboreal (e.g. cavities) sites. Furthermore, fat individuals are less agile in their ability to catch prey such as rodents.

Both martens avoid open habitats (e.g. meadows or fields), likely to reduce predation risk from larger predators, like fox, lynx and dog (*Canis lupus familiaris*). Stone marten preferred shrubs, ecotone areas and brushwoods during movement or dispersal [28]. Pine martens avoid open areas, like meadows and even clear-cuts in the forest, to an even greater extent [76, 96], and they use a more arboreal way of moving in tree crowns than stone marten [28]. The less arboreal movement pattern of stone martens may expose it to higher predation pressure in the forest. In villages, the lower abundance of predators (especially foxes) may reduce predation risk for stone martens and buildings, wooden fences, piles of wood and other human-related constructions may create safe ‘movement corridors’ for stone marten.

Whatever the mechanism, the near complete spatial niche segregation between these martens appears to facilitate the coexistence of these species at our site. Other carnivores occupying similar niches and possessing similar morphological features have been observed excluding each other if one is larger than the other, which often causes the disappearance of the smaller one. For example, European and American mink (*Mustela lutreola* and *Neovision vision*) are characterized by similar morphology and ecology and both species occupy semi-aquatic habitats. The invasion of American mink in Europe caused drastic declines of European mink due to interspecific competition [112–114]. Likewise, the smaller arctic fox (*Vulpes lagopus*) is excluded from more productive habitats by the larger red fox [18, 19] and the least weasel (*Mustela nivalis*) can be excluded from habitats with high vole densities by the two times larger stoat (*Mustela erminea*) through interference competition [115].

In conclusion, the near complete habitat niche segregation of the two similar marten species facilitates their coexistence in our study area. We suggest that spatial niche segregation between these species may be influenced by their adaptations to urban areas, to avoid the effects of cold climate, food preferences and/or behavioral predator avoidance, and not competitive interactions. Climate mediates interactions between food abundance and predation pressure affecting large variation of habitat use and preference of stone marten in its geographic range. Larger stone martens selected habitats with more stable food availability which influenced its better body condition, but this may also reduce its arboreal behavior. Ability to use arboreal resting sites by large stone martens may be limited, therefore it selects villages with a greater abundance of insulated and safe resting sites in human constructions. These results are the first indication of the importance of anthropogenic habitats for stone martens living in the most northern portion of its range and suggest that human-altered habitat may have facilitated its survival within a large forest complex, in coexistence with pine martens.

## Supporting Information

S1 Table Differences in Akaike’s information criterion scores (ΔAIC_c_) and AIC_c_ weights for candidate body weight and body length models developed for stone and pine marten populations of north-eastern Poland. (DOCX)

S2 Table Differences in Akaike’s information criterion scores (ΔAIC_c_) and AIC_c_ weights for candidate activity models developed for stone and pine marten populations of north-eastern Poland. (DOCX)

S3 Table Differences in Akaike’s information criterion scores (ΔAIC_c_) and AIC_c_ weights for candidate duration of activity models developed for stone and pine marten populations of north-eastern Poland. (DOCX)
