# Supplementary material for: Spatial Niche Segregation of Sympatric Stone Marten and Pine Marten – Avoidance of Competition or Selection of Optimal Habitat?
Source: PLoS One. 2015 Oct 7;10(10):e0139852. doi: 10.1371/journal.pone.0139852 (PMC4596623; doi:10.1371/journal.pone.0139852)
Supplement: S1 Table — (DOCX) [file pone.0139852.s001.docx]

| **No** | **Covariates** | **AICc** | **∆AIC_c_** | **weight** |
| --- | --- | --- | --- | --- |
| Body weight | | | | |
| 1 | Species, Sex | - 70.1 | 0.00 | 0.618 |
| 2 | Species, Sex, Species*Sex | - 69.1 | 0.96 | 0.382 |
| 3 | Sex | - 40.3 | 29.79 | 0.000 |
| Body length | | | | |
| 1 | Species, Sex, Species*Sex | 314.6 | 0.00 | 0.579 |
| 2 | Species, Sex | 316.5 | 1.91 | 0.223 |
| 3 | Sex | 316. | 2.15 | 0.198 |
| 4 | Species | 348.8 | 34.21 | 0.000 |
