# Supplementary material for: Spatial Niche Segregation of Sympatric Stone Marten and Pine Marten – Avoidance of Competition or Selection of Optimal Habitat?
Source: PLoS One. 2015 Oct 7;10(10):e0139852. doi: 10.1371/journal.pone.0139852 (PMC4596623; doi:10.1371/journal.pone.0139852)
Supplement: S2 Table — (DOCX) [file pone.0139852.s002.docx]

| **No** | **Covariates** | **AICc** | **∆AIC_c_** | **weight** |
| --- | --- | --- | --- | --- |
| 1 | Hour, Season, Sex, Species | 29866.6 | 0.00 | 0.829 |
| 2 | Hour, Season, Sex | 29869.8 | 3.15 | 0.171 |
| 3 | Hour, Season, Species | 29890.1 | 23.49 | 0.000 |
