# Supplementary material for: Spatial Niche Segregation of Sympatric Stone Marten and Pine Marten – Avoidance of Competition or Selection of Optimal Habitat?
Source: PLoS One. 2015 Oct 7;10(10):e0139852. doi: 10.1371/journal.pone.0139852 (PMC4596623; doi:10.1371/journal.pone.0139852)
Supplement: S3 Table — (DOCX) [file pone.0139852.s003.docx]

| **No** | **Covariates** | **AICc** | **∆AIC_c_** | **weight** |
| --- | --- | --- | --- | --- |
| 1 | Species, Sex, Season, Species*Season | 261.7 | 0.00 | 0.471 |
| 2 | Species, Sex, Season, Species:Season, Sex*Season | 262.6 | 0.95 | 0.293 |
| 3 | Species, Season, Species*Season | 263.1 | 1.38 | 0.236 |
| 4 | Species, Sex, Season | 285.0 | 23.29 | 0.000 |
